# Red Blood Cell Distribution Width (RDW) Predicts COVID-19 Severity: A Prospective, Observational Study from the Cincinnati SARS-CoV-2 Emergency Department Cohort

**DOI:** 10.3390/diagnostics10090618

**Published:** 2020-08-21

**Authors:** Brandon Michael Henry, Justin Lee Benoit, Stefanie Benoit, Christina Pulvino, Brandon A. Berger, Maria Helena Santos de Olivera, Christopher A. Crutchfield, Giuseppe Lippi

**Affiliations:** 1 Cardiac Intensive Care Unit, The Heart Institute, Cincinnati Children’s Hospital Medical Center, Cincinnati, OH 45229, USA; 2 Department of Emergency Medicine, University of Cincinnati, Cincinnati, OH 45221, USA; benoitjn@ucmail.uc.edu (J.L.B.); christina.pulvino@uc.edu (C.P.); bergerbd@ucmail.uc.edu (B.A.B.); 3 Division of Nephrology and Hypertension, Cincinnati Children’s Hospital Medical Center, Cincinnati, OH 45229, USA; stefanie.benoit@cchmc.org; 4 Department of Pediatrics, University of Cincinnati, College of Medicine, Cincinnati, OH 45229, USA; 5 Department of Statistics, Federal University of Parana, Curitiba 80060-000, Brazil; mariaholiveira34@gmail.com; 6 Department of Pathology & Laboratory Medicine, University of Cincinnati, College of Medicine, OH 45219, USA; crutchcs@ucmail.uc.edu; 7 Section of Clinical Biochemistry, Department of Neuroscience, Biomedicine and Movement, University of Verona, 37129 Verona, Italy; giuseppe.lippi@univr.it

**Keywords:** diagnostics, hematology, red blood cells, anisocytosis, infections, acute kidney injury

## Abstract

Since previous evidence has demonstrated that red blood cell distribution width (RDW) may be a useful prognostic parameter in many critical illnesses and infectious diseases, we investigated the utility of RDW for monitoring patients with coronavirus disease 2019 (COVID-19). The study population consisted of 49 COVID-19 patients, including 16 (32.6%) with severe illness, 12 (24.5%) with severe acute kidney injury (AKI), and 8 (16.3%) requiring renal replacement therapy (RRT). The predictive value of blood tests, performed during emergency department evaluation, was then addressed. A progressive increase of RDW was observed with advancing COVID-19 severity. The area under the curve (AUC) of RDW was 0.73 for predicting severe illness, 0.80 for severe AKI, and 0.83 for RRT, respectively. In multivariate analysis, elevated RDW was associated with 9-fold and 16-fold increased odds of severe COVID-19 and AKI, respectively. The results of this study suggest that RDW should be part of routine laboratory assessment and monitoring of COVID-19.

## 1. Introduction

The identification of clinical, demographic, and laboratory factors predictive of clinical deterioration and prognosis is a top research priority in the ongoing coronavirus disease 2019 (COVID-19) pandemic [1]. This severe infectious disease, which is caused by severe acute respiratory syndrome coronavirus 2 (SARS-CoV-2), is frequently characterized by the need for mechanical ventilation and intensive care, with a cumulative intensive care unit (ICU) mortality rate that has been reported to be as high as 40% [2].

The concept of using laboratory investigations for early risk stratification is based on a rationale that predictive biomarkers of severe disease would enable the timely identification of patients at higher risk of progression towards unfavorable outcomes (respiratory distress, multiple organ failure, and even death). This may enable an earlier and more appropriate therapeutic intervention, thus focusing the allocation of limited healthcare resources on patients whom would receive the greatest benefits [3]. Nonetheless, the significant variability in severity of illness and patient heterogeneity, as well as overwhelmed laboratories with little additional resources for special and complex assays, makes this a challenging pursuit [4].

Red blood cell distribution width (RDW) is a readily available laboratory parameter reported on the complete blood count (CBC) with differential by many modern hematology analyzers. In short, this measure reflects the extent of anisocytosis, a condition characterized by pronounced heterogeneity in the volume of circulating erythrocytes [5]. In critically ill patients with sepsis, baseline RDW has been shown to be a significant and independent predictor of mortality [6], while another recent study reported elevated RDW was associated with decreased ventilator free days in the intensive care unit [7]. Nevertheless, only a few studies have explored the role of this potentially important laboratory parameter in COVID-19, or examined its utility for predicting the clinical outcome in patients with SARS-CoV-2 infection.

In this report, we performed an analysis of red blood cell (RBC) indices across the spectrum of COVID-19 severity and analyzed the diagnostic performance of RDW for predicting development of severe disease, acute kidney injury (AKI), and the need for renal replacement therapy (RRT). AKI and RRT were selected based on their association with a poor prognosis in patients with COVID-19 [8].

## 2. Materials and Methods

This prospective, observational study was based on a Cincinnati COVID-19 Emergency Department (ED) Cohort. Adults (≥18 years of age) presenting to the ED of the University of Cincinnati Medical Center with symptoms suggestive of COVID-19 and undergoing a clinically required blood draw for routine work-up were enrolled via an IRB-approved waiver of informed consent. Final inclusion in the study cohort was dependent on positive result for SARS-CoV-2 based on a routine reverse transcription polymerase chain reaction (RT-PCR) test performed on nasopharyngeal swabs. Patients lacking routinely performed CBC with differential prior to ED disposition were excluded from the study.

Blood samples were obtained as part of standard of care during the index visit to the ED. The CBC with differential was performed using a Beckman UniCel DxH 800 Coulter Cellular Analysis System (Brea, CA, USA). The technical features as well as the analytical performance of this hematological analyzer have been comprehensively described elsewhere [9]. Serum creatinine assays were performed using a kinetic alkaline picrate (modified Jaffe) method on either a Beckman Coulter AU480 Chemistry Analyzer (Brea, CA, USA) or a Beckman Coulter AU5822 Chemistry Analyzer (Brea, CA, USA). High-Sensitivity C-Reactive Protein (CRP) was measured using particle enhanced immunonephelometry on a Behring Nephelometer (BN) II System (Siemens Medical Solutions USA, Inc., Malvern, PA, USA). Ferritin was measured using a latex immunoassay on BN II System. Patient characteristics data, co-morbidities, presenting vital signs, laboratory data (including RBC indices), and clinical outcome were extracted from electronic medical records (EMR) and recorded into a REDCap (Research Electronic Data Capture) database. RDW data were recorded as RDW-coefficient of variation (RDW-CV). Data extraction was performed by an ED physician, with select records checked for accuracy by a second physician. Data on the clinical course of patients admitted from the ED were collected through discharge/death, while data on the clinical course of patients discharged following the index ED visit were monitored for 30 days.

The primary outcome of this study was COVID-19 peak severity within 30 days of index ED visit. Mild disease was defined as not being hospitalized for COVID-19 or for its complications. Moderate disease was defined as hospitalized with or without supplemental oxygen by mask or nasal cannula. Severe disease was defined as any level of respiratory support at or beyond non-invasive ventilation or high flow oxygen devices or illness requiring ICU admission. The secondary outcomes were severity at ED disposition and development of severe acute kidney injury during hospitalization defined as Kidney Disease Improving Global Outcomes (KDIGO) Stage 2 and 3 assessed using serum creatinine criteria [10]. The tertiary outcome was need for renal replacement therapy (RRT).

Categorical data were reported as absolute number (*n*) and relative frequency (%), whilst continuous variables were reported as median and interquartile range (IQR). The diagnostic performance of RDW for predicting the clinical outcomes was assessed using receiver operating characteristics (ROC), with calculation of the area under the curve (AUC) and its 95% confidence interval (95% CI). Logistic regression was performed to estimate the effect of elevated RDW (≥14.5%, based on ROC analysis) on primary outcome of severe disease, adjusting for demographic characteristics from Table 1. Further variable selection and model improvement was performed using the stepwise algorithm. The same process was repeated for the secondary outcome of severe AKI (KDIGO 2+3). Final logistic models were used to calculate adjusted odds ratios as well as the corresponding 95% Wald confidence intervals. Statistical analyses were carried out with Prism 8 (GraphPad Software, San Diego, CA, USA) and R version 3.6.1 (R Foundation for Statistical Computing, Vienna, Austria), and statistical significance was set at *p* < 0.05.

## 3. Results

### 3.1. Partient Characteristics and Outcomes

A total of 52 consecutive adults with laboratory-confirmed COVID-19 were initially included in this study. Three patients, discharged at ED disposition with mild disease, had to be excluded due to the lack of clinically ordered CBC. Therefore, the final cohort consisted of 49 patients, whose characteristics are summarized in Table 1. With respect to disease, a significant median age difference increase was observed between each progressive stage of severity (*p* = 0.002). Diabetes, hypertension, and chronic obstructive pulmonary disease were observed more frequently in patients with moderate and severe disease as opposed to those with mild illness. At ED disposition, a total of 16 (32.7%) patients had mild disease and were discharged for outpatient treatment (none of which were hospitalized within 30 days), 27 (55.1%) had moderate disease and were admitted, and 6 (12.2%) had severe disease requiring immediate ICU admission. Throughout the entire course of hospitalization, 10 patients with moderate disease progressed to severe disease. Thus, a total of 32.6% (*n* = 16) patients reached the severe form of COVID-19. A total of 24.5% (*n* = 12) patients reached the secondary outcome of severe AKI (KDIGO 2+3), while 16.3% (*n* = 8) of patients required renal replacement therapy.

### 3.2. Red Blood Cell Indices and RDW

Analysis of RBC indices by severity at ED disposition and peak 30-day severity are shown in Table 2. For ED disposition, patients with severe COVID-19 had significantly lower hematocrit and hemoglobin values, compared to those with mild/moderate disease (both *p* < 0.001), whilst a progressive increase of RDW values was observed with advancing severity (*p* < 0.001). At maximum 30-day severity, similar findings were observed. Hematocrit and hemoglobin trended significantly lower with progressive severity (*p* = 0.002 and *p* = 0.017, respectively). RDW progressively increased with progressive severity (*p* = 0.001).

### 3.3. Diagnostic Performance of RDW

**Figure 1:**
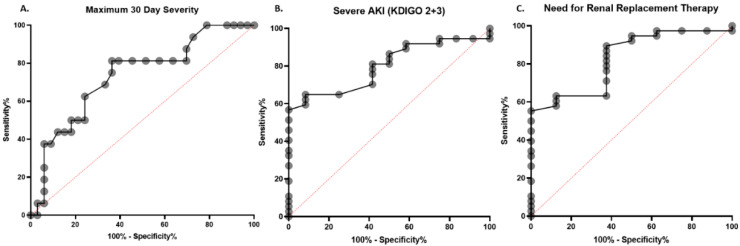
Receiver operating characteristics (ROC) curve of red blood cell distribution width (RDW) for the prediction of (**A**) progression to severe COVID-19 within 30 days of Emergency Department presentation (AUC: 0.73); (**B**) severe acute kidney injury (KDIGO 2 + 3) (AUC: 0.80); and (**C**) need for renal replacement therapy (AUC: 0.83). Dashed red line represents the line of non-discrimination.

An ROC was generated for RDW at time of initial ED evaluation for predicting peak disease severity within 30 days of index ED visit (Figure 1A). The RDW AUC for the severe form of COVID-19 was 0.73 (95% CI, 0.58–0.88; *p* = 0.008). Analysis of ROC data determined that an RDW cut-off of ≥14.5% was associated with 0.81 sensitivity and 0.64 specificity, respectively. The ROC generated for predicting severe AKI (KDIGO 2+3) and need for RRT within 30 days of index ED visit is shown in Figure 1B,C. The RDW AUC for severe AKI (KDIGO 2+3) was 0.80 (95% CI, 0.68–0.92; *p* = 0.002), with ≥14.6% cutoff displaying 0.65 sensitivity and 0.92 specificity. The RDW AUC for need of RRT was 0.83 (95% CI, 0.68–0.97; *p* = 0.004), with ≥14.6% cutoff displaying 0.63 sensitivity and 0.88 specificity.

### 3.4. RDW as Predictor of Disease Severity and Severe AKI

The results of multivariable logistic regression for above-normal RDW (i.e., ≥14.5%) as independent predictor of peak 30-day disease severity and severe AKI (KIDGO 2+3) are shown in Table 3. After controlling for confounders, elevated RDW was associated with 9-fold increased odds of severe COVID-19 (*p* = 0.046) and 16-fold increased odds of severe AKI (KDIGO 2+3) (*p* = 0.0143), respectively.

## 4. Discussion

Due to frequent progression of COVID-19 to severity requiring ventilatory support and intensive care, the ongoing COVID-19 pandemic has led to a considerable demand for hospital beds, thus overwhelming the responsiveness of many healthcare systems worldwide. In this critical reality, it has now become clear that accurate and timely prognostic information is needed to improve patient management with limited healthcare resources [11]. As such, the availability of prognostic laboratory tests, which are inherently characterized by low invasiveness, high yield, and quick turnaround time, compared to other diagnostic investigations, may be highly valuable tools for risk stratification [12].

Among the multitude of laboratory parameters that have been found to have a significant prognostic value, RDW has gained significant attention during the past decade due to its capability to efficiently predict the risk of death in the general population [13], in patients with non-cardiovascular critical illness [14], sepsis [15], pneumonia, and other respiratory tract infections [16]. It is not surprising that our results demonstrate RDW as a significant and independent predictor of both disease severity and kidney injury in patients with SARS-CoV-2 infection. Notably, after controlling for confounders, an increased value of this laboratory parameter was associated with 9.2- and 16.0-fold enhanced risk for predicting severe illness and AKI, with respective prognostic accuracies as high as 73% and 80%.

In this study, we found that patients with mild illness at the time of index ED visit had a median RDW (13.5%) within the normal range (11.6–14.6%) [5], while those with moderate illness had a median RDW slightly above normal (14.8%) and those with severe disease had a median substantially above normal (17.7%). Our results are comparable to patients with other respiratory illnesses. Lee et al. [16] reported RDW values at index ED visit in patients hospitalized with community acquired pneumonia (CAP), finding a median RDW value of 14.1% (IQR: 13.3–15.2%), which is similar to the median RDW value of 14.8% (13.9–16.7%) of the moderate COVID-19 patients (i.e., hospitalized but non-severe cases). The authors also found that elevated RDW at index ED visit was associated with poor outcomes in CAP, including 30-date mortality, length of hospital stay, and need for vasopressors [16]. Moreover, our findings are in agreement with other data published in patients with other forms of ARDS not attributable to SARS-CoV-2 infection [17], as well as in an earlier publication on COVID-19 patients, characterized by a different setting and patient cohort. Regarding this latter study, Wang et al. measured RDW in 45 COVID-19 patients with moderate and severe illness admitted to the Jingzhou Central Hospital from over a nearly 20-day period [18]. The RDW value was found to be significantly higher in patients with severe COVID-19 than in those with milder form of disease. Moreover, both RDWs were found to be significant predictors of severe illness, displaying between 65 and 76% diagnostic accuracy.

Multiple theoretically viable hypotheses can be made to justify the potential prognostic role of anisocytosis in COVID-19, encompassing direct cytopathic injury due to infection of circulating erythrocytes or their bone marrow precursors, indirect erythrocyte damage consequent to hemolytic anemia or intravascular coagulopathy, and profound perturbation of iron metabolism due to the sustained inflammatory response. All of these conditions would ultimately contribute to deranged erythrocyte biology and explain their considerable size variation within the circulation.

With respect to the potential for direct cytopathic injury on RBC, a recent study found that SARS-CoV-2 infection generates important structural changes in membrane homeostasis of circulating RBCs, both at the lipid and protein level [19]. More specifically, the authors of this investigation found that circulating erythrocytes of COVID-19 patients displayed substantially enhanced expression of glycolytic intermediates, combined with significant degree of oxidative damages and consequent fragmentation of proteins such as spectrin beta, ankyrin, and N-terminal cytosolic domain of band 3 (AE1). Abnormalities of lipid metabolism could also be identified, encompassing especially acyl-carnitines, sphingolipids, along with short- and medium-chain saturated fatty acids. A direct bone marrow injury secondary to SARS-CoV-2 infection has also been described in several reports, mostly manifesting as hemophagocytosis, with clear evidence of erythrophagocytic phenomena characterized by macrophages engulfed with both erythroblasts and mature erythrocytes [20].

A coexistent indirect injury may also help to explain the dramatic derangement of erythrocyte biology in patients with SARS-CoV-2 infection. First, there have been reported cases of autoimmune hemolytic anemia that have been associated with SARS-CoV-2 infection [21], a phenomenon that has been mostly attributed to the high molecular mimicry between the spike protein of SARS-CoV-2 and the protein ankyrin 1 present at RBC surface [22]. Intravascular coagulopathy, either localized to the lung parenchyma or disseminated, is also common in COVID-19 patients developing severe or critical illness [23]. The development of micro- and macro-thrombi in many blood vessels is a well-known cause of erythrocyte injury, which would contribute to the presence of RBCs with many morphological abnormalities (e.g., especially schistocytes) and large size heterogeneity within the circulation [24]. Moreover, it is possible that such microthrombi could perpetuate AKI, which could lead to disrupted production of erythropoietin, thus exacerbating anisocytosis and/or anemia in patients with COVID-19.

Finally, the presence of a sustained inflammatory state is another mechanism that could trigger and sustain the marked anisocytosis observed in COVID-19 patients with severe illness in our study. Erythrocyte biology is strongly dependent on iron metabolism, and it is now universally clear that many inflammatory conditions are associated with markedly elevated erythrocyte size variability. This is attributable to a kaleidoscope of metabolic derangements occurring in concomitance with systemic inflammation, encompassing reduced iron absorption and availability, impaired erythropoiesis with accelerated release of larger RBCs into the circulation, as well as oxidative erythrocyte injury, which would ultimately impair their flexibility, size, and morphology [25]. In fact, elevated ferritin has become a prominent biomarker in COVID-19, with elevations associated with development of severe disease [12].

This study was limited by a rather small sample size and single-center design. Many variables, including age, sex, race, and co-morbidities, may influence RDW value and potentially bias results in a small sample; however, we controlled for such confounders in a multivariate regression analysis. Future investigations should be performed on a variety of diverse cohorts, as differences in laboratory abnormalities between different global geographical regions have been reported [26]. Moreover, while we provide evidence of utility for RDW as a single-point prognostic biomarker at time of ED evaluation, future single and multi-center studies should be performed to assess the value of RDW at predicting clinical deterioration over course of hospitalization in patients with COVID-19, as well to evaluate the prognostic value of RDW at other important time points, such as ICU admission.

## 5. Conclusions

The results of this investigation support the assumption that RDW assessment should be part of the routine panel of laboratory tests offered for COVID-19 patient monitoring, along with other hematologic, biochemical, hemostasis, and immunochemical tests [27].

## Figures and Tables

**Table 1:** Characteristics of Cincinnati COVID-19 Emergency Department Cohort (*n* = 49).

Variable	Maximum 30-Day Severity			
	Mild (*n* = 16)	Moderate (*n* = 17)	Severe (*n* = 16)	*p*-Value
Male: *n* (%)	11 (68.8%)	8 (44.4%)	10 (62.5%)	0.425
Age (years): median (IQR)	44 (32–50)	59 (39–68)	66 (53–71)	0.002
Body Mass Index: median (IQR)	28 (25–33)	26 (25–36)	29 (25–32)	0.886
Race/Ethnicity: *n* (%)				
Asian	0 (0%)	1 (5.9%)	0 (0%)	0.093
Black	4 (25.0%)	10 (58.8%)	7 (41.2%)	
Hispanic	9 (56.2%)	3 (17.6%)	5 (31.3%)	
White	1 (6.3%)	3 (17.6%)	4 (25.0%)	
Multiracial/Other	2 (12.5%)	0 (0%)	0 (0%)	
Comorbidities: *n* (%)				
Coronary Artery Disease	0 (0%)	4 (23.5%)	3 (18.8%)	0.128
Heart Failure	0 (0%)	4 (23.5%)	5 (31.3%)	0.059
Hypertension	3 (18.8%)	11 (64.7%)	11 (68.8%)	0.007
Hyperlipidemia	2 (12.5%)	5 (29.4%)	5 (31.3%)	0.395
Diabetes	3 (18.8%)	12 (70.6%)	5 (31.3%)	0.007
Chronic Obstructive Pulmonary Disease	0 (0%)	2 (11.8%)	6 (37.5%)	0.013
Chronic Kidney Disease	0 (0%)	3 (17.6%)	3 (18.8%)	0.191
Chronic Liver Disease	1 (0%)	2 (11.8%)	4 (25.0%)	0.296
Cerebrovascular Disease	0 (0%)	3 (17.6%)	4 (25.0%)	0.115
Cancer	0 (0%)	1 (5.9%)	3 (18.8%)	0.148
Obesity	5 (31.3%)	8 (44.4%)	5 (31.3%)	0.551
Current Smoker	4 (25.0%)	4 (23.5%)	5 (31.3%)	0.869
Former Smoker	1 (6.3%)	3 (17.6%)	4 (25.0%)	0.351
Labs at ED * Presentation: Median (IQR)				
White Blood Cell Count (×10^3^/mm^3^)	5.7 (4.6–8.2)	7.0 (5.7–9.8)	6.8 (5.2–9.6)	0.442
Neutrophil Count (×10^3^/mm^3^)	3.8 (2.8–6.2)	4.8 (4.0–8.0)	5.2 (3.9–8.8)	0.034
Lymphocyte Count (×10^3^/mm^3^)	0.8 (0.5–1.6)	1.0 (0.8–1.4)	0.8 (0.6–1.2)	0.332
Platelet Count (×10^3^/mm^3^)	193.0 (164.8–238.0)	208.0 (155.0–275.3)	211.5 (152.5–346.8)	0.710
C-Reactive Protein (mg/dL)	1.5 (0.7–7.5)	8.6 (2.8–15.8)	4.8 (2.7–12.2)	0.108
Ferritin (ug/L)	352.0 (122.0–1088.0)	251.0 (101.5–995.5)	1032.0 (232.5–1475.0)	0.1198
Days from symptom onset to ED presentation: median (IQR)	6 (3–7)	7 (5–10)	5 (1–10)	0.551

* ED—emergency department, IQR—interquartile range. *p*-value calculated using Kruskal–Wallis test or Chi-square test.

**Table 2:** Red Blood Cell Indices in Patients with COVID-19.

Variable	ED Disposition Severity				Maximum Severity within 30 Days of Presentation			
	Mild (*n* = 16)	Moderate (*n* = 27)	Severe (*n* = 6)	*p*-Value	Mild (*n* = 16)	Moderate (*n* = 17)	Severe (*n* = 16)	*p*-Value
Hematocrit (%)	43.9 (40.4–45.4)	37.5 (34.3–41.4)	35.1 (24.7–42.0)	<0.001	43.9 (40.4–45.4)	38.5 (34.8–41.3)	37.1 (31.9–42.1)	0.002
Hemoglobin (g/dL)	14.8 (14.0–15.4)	12.6 (11.2–13.7)	11.1 (8.4–13.9)	<0.001	14.1 (13.7–15.2)	12.3 (11.3–13.4)	12.5 (10.1–14.0)	0.017
Mean corpuscular volume (MCV) (fL)	86.4 (82.9–89.1)	85.9 (81.1–88.3)	86.7 (81.5–94.4)	0.639	86.4 (82.9–89.1)	85.8 (81.8–87.6)	86.0 (81.1–90.5)	0.685
Mean corpuscular hemoglobin (MCH) (pg/cell)	29.7 (28.7–30.6)	27.7 (26.5–30.1)	29.4 (27.1–31.1)	0.236	29.7 (28.7–30.6)	27.3 (26.7–29.9)	29.3 (26.7–31.1)	0.244
Mean corpuscular hemoglobin concentration (MCHC) (g/DL)	34.4 (33.3–34.7)	32.8 (31.4–34.0)	33.3 (31.4–34.6)	0.049	34.4 (33.3–34.7)	32.3 (31.6–33.9)	33.5 (32.1–34.3)	0.035
Red Blood Cell Distribution Width (RDW-CV) (%)	13.5 (13.1–13.9)	14.8 (13.9–16.7)	17.7 (14.9–19.48)	<0.001	13.5 (13.1–13.8)	14.8 (13.8–16.7)	16.0 (14.6–18.7)	0.001

**Table 3:** Results of multivariable logistic regression for RDW prediction of severe form of COVID-19 and severe acute kidney injury (AKI) (KDIGO 2 + 3) within 30 days of index Emergency Department visit.

**Severe COVID-19**				
**Variable**	**Estimate**	**Std Error**	**OR (95% CI)**	***p*-Value**
Age	0.109	0.042	1.12 (1.03–1.21)	0.010
Sex (Male)	1.856	0.974	6.40 (0.95–43.12)	0.057
Race (Hispanic)	2.485	1.311	12 (0.92–156.66)	0.058
Race (White)	−0.106	1.143	0.90 (0.1–8.45)	0.926
Elevated RDW	2.220	1.114	9.20 (1.04–81.74)	0.046
**Severe AKI (KDIGO)**				
**Variable**	**Estimate**	**Std Error**	**OR (95% CI)**	***p*-Value**
BMI	−0.134	0.066	0.87 (0.7–0.99)	0.043
Elevated RDW	2.774	1.133	16.03 (1.74–147.6)	0.014
